# Exploring the Antimycobacterial Potential of Podocarpusflavone A from *Kielmeyera membranacea*: In Vitro and In Vivo Insights

**DOI:** 10.3390/ph17121560

**Published:** 2024-11-21

**Authors:** Marlon Heggdorne de Araujo, Salomé Muñoz Sánchez, Thatiana Lopes Biá Ventura Simão, Natalia Nowik, Stella Schuenck Antunes, Shaft Corrêa Pinto, Davide Sorze, Francesca Boldrin, Riccardo Manganelli, Nelilma Correia Romeiro, Elena B. Lasunskaia, Fons J. Verbeek, Herman P. Spaink, Michelle Frazão Muzitano

**Affiliations:** 1 Laboratório de Produtos Bioativos (LPBio), Instituto de Ciências Farmacêuticas, Universidade Federal do Rio de Janeiro, Campus Macaé, Macaé 27930-560, RJ, Brazil; marlon.heggdorne@gmail.com (M.H.d.A.); stellantunes@yahoo.com.br (S.S.A.); shaftcp@yahoo.com.br (S.C.P.); 2 Department of Animal Sciences and Health, Institute of Biology (IBL), Leiden University, 2333 BE Leiden, The Netherlands; s.munoz.sanchez@biology.leidenuniv.nl (S.M.S.); h.p.spaink@biology.leidenuniv.nl (H.P.S.); 3 Laboratório de Biologia do Reconhecer (LBR), Centro de Biociências e Biotecnologia, Universidade Estadual do Norte Fluminense Darcy Ribeiro, Campos dos Goytacazes 28013-602, RJ, Brazil; thativentura@yahoo.com.br (T.L.B.V.S.); elena@uenf.br (E.B.L.); 4 Laboratório Integrado de Computação Científica (LICC), Universidade Federal do Rio de Janeiro, Campus Macaé, Macaé 27930-560, RJ, Brazil; nelilmaromeiro@gmail.com; 5 Department of Molecular Medicine, University of Padova, 35121 Padova, Italy; davide.sorze@studenti.unipd.it (D.S.); francesca.boldrin@unipd.it (F.B.); riccardo.manganelli@unipd.it (R.M.); 6 Leiden Institute of Advanced Computer Science, Leiden University, 2333 CA Leiden, The Netherlands; f.j.verbeek@liacs.leidenuniv.nl

**Keywords:** biflavone, podocarpusflavone A, zebrafish, tuberculosis, *Kielmeyera*, cytokines, toxicity

## Abstract

**Background/Objectives**: Tuberculosis (TB) is one of the leading infectious causes of death worldwide, highlighting the importance of identifying new anti-TB agents. In previous research, our team identified antimycobacterial activity in *Kielmeyera membranacea* leaf extract; therefore, this study aims to conduct further exploration of its potential. **Methods**: Classical chromatography was applied for fractionation and spectrometric techniques were utilized for chemical characterization. For in vitro tests, samples were assessed against *Mycobacterium tuberculosis* and *Mycobacterium marinum*. The toxicity and efficacy of active samples were evaluated in vivo using different zebrafish models. Chemogenomics studies were applied to predict the isolated active compound’s potential mode of action. **Results**: We performed fractionation of *K. membranacea* ethanolic extract (EE) and then its dichloromethane fraction (DCM), and the biflavonoid podocarpusflavone A (PCFA) was isolated and identified as a promising active compound. The EE and PCFA were found to be non-toxic to zebrafish larvae and were able to inhibit *M. tuberculosis* growth extracellularly. Additionally, PCFA demonstrated antimycobacterial activity within infected macrophages, especially when combined with isoniazid. In addition, the EE, DCM, and PCFA have shown the ability to inhibit *M. marinum’s* growth during in vivo zebrafish larvae yolk infection. Notably, PCFA also effectively countered systemic infection established through the caudal vein, showing a similar inhibitory activity profile to rifampicin, both at 32 µM. A reduction in the transcriptional levels of pro-inflammatory cytokines confirmed the infection resolution. The protein tyrosine phosphatase B (PtpB) of *M*. *tuberculosis*, which inhibits the macrophage immune response, was predicted as a theoretical target of PCFA. This finding is in agreement with the higher activity observed for PCFA intracellularly and in vivo on zebrafish, compared with the direct action in *M. tuberculosis*. **Conclusions**: Here, we describe the discovery of PCFA as an intracellular inhibitor of *M. tuberculosis* and provide evidence of its in vivo efficacy and safety, encouraging its further development as a combination drug in novel therapeutic regimens for TB.

## 1. Introduction

Tuberculosis (TB) is an infectious disease that affects the lungs and is caused by *Mycobacterium tuberculosis*. Globally, in 2023, an estimated 10.8 million people fell ill with TB, resulting in 1.25 million deaths [1]. Additionally, drug-resistant TB remains a significant public health threat. From 2018 to 2023, over 8.2 million people worldwide enrolled in treatment for multidrug-resistant or rifampicin-resistant TB (MDR/RR-TB). In 2023 alone, 188,666 cases were identified, including 159,684 cases of MDR/RR-TB and 28,982 cases of pre-extensive drug-resistant (pre-XDR) TB or extensively drug-resistant TB (XDR-TB). Current data indicate a treatment success rate for RR-TB between 50 and 75%, emphasizing the critical need to study new, effective, and safe anti-TB drugs [1].

Natural products are part of an essential strategy for the development of new drugs for the treatment of TB [2,3]. In this scenario, the coastal ecosystem of the restinga, which belongs to the Atlantic Forest biome, is a recognized biodiversity hotspot. Its extreme environmental conditions can lead to diverse production of secondary metabolites. Among the plants that occur in restingas, *Kielmeyera* species (*Calophyllaceae*) can be highlighted for their antimicrobial potential against bacteria [4,5,6] and fungi [7], in addition to their antiglycation [8], anticancer [9], and anxiolytic [10] activities. Regarding their chemical composition, procyanidins [8], coumarins [9], xanthones [5], long-chain fatty acid compounds [11], and sesquiterpenes [6] have been described. However, there is limited research on *K. membranacea* Casar., with published information mainly addressing its botanical and ecological characteristics [12], except for a prior screening study conducted by our research group, which identified the in vitro antimycobacterial activity of *Kielmeyera membranacea* [13]. In this previous study, different plant species collected at the Jurubatiba National Park, a protected restinga area in the Northeast of Rio de Janeiro (Brazil), were evaluated against *Mycobacterium bovis* BCG. *Kielmeyera membranacea* was among the most active species, especially its dichloromethane fraction, which inhibited the extracellular growth of *M. bovis* BCG and *M. tuberculosis* H37Rv strains [13]. Therefore, considering the urgent need for new anti-TB compounds, the present study aimed to investigate the *Kielmeyera membranacea* extract, fraction, and isolated compound, including in vitro and in vivo experiments.

Studies of anti-TB activity with bioactive natural products using in vivo models are still scarce, both due to the difficulty in simulating the human pathology using animal models and because in vivo experiments require a more significant amount of samples to treat the groups of animals. In this context, the zebrafish (*Danio rerio*) model uses compounds economically while maintaining the experimental complexity of in vivo models [14]. *Mycobacterium marinum* is a natural pathogen of zebrafish and can cause granulomatous lesions with host tissue necrosis [15], being genetically related to the *M. tuberculosis* complex. In zebrafish, it causes a disease that resembles TB in humans [16], allowing investigators to quickly assess the inhibitory effects of natural products on mycobacterial growth [16,17] and also to evaluate toxic effects during larval development [18].

In view of the importance of conducting research to find new anti-TB compounds, the present study focused on evaluating the antimycobacterial activity of podocarpusflavone A, isolated from *Kielmeyera membranacea,* in vitro and in vivo through the *M. marinum*-infected zebrafish larval model. In addition, the antimycobacterial activity and the toxicological profile of the ethanol extract (EE) of *Kielmeyera membranacea* were investigated in vivo. Therefore, this original study provides important and robust data regarding the in vitro and in vivo antimycobacterial activity of podocarpusflavone A for the first time; in addition, it contributes to the knowledge about the species *Kielmeyera membranacea*.

## 2. Results and Discussion

### 2.1. Chemical Study of Kielmeyera Membranacea

**Figure 1:**
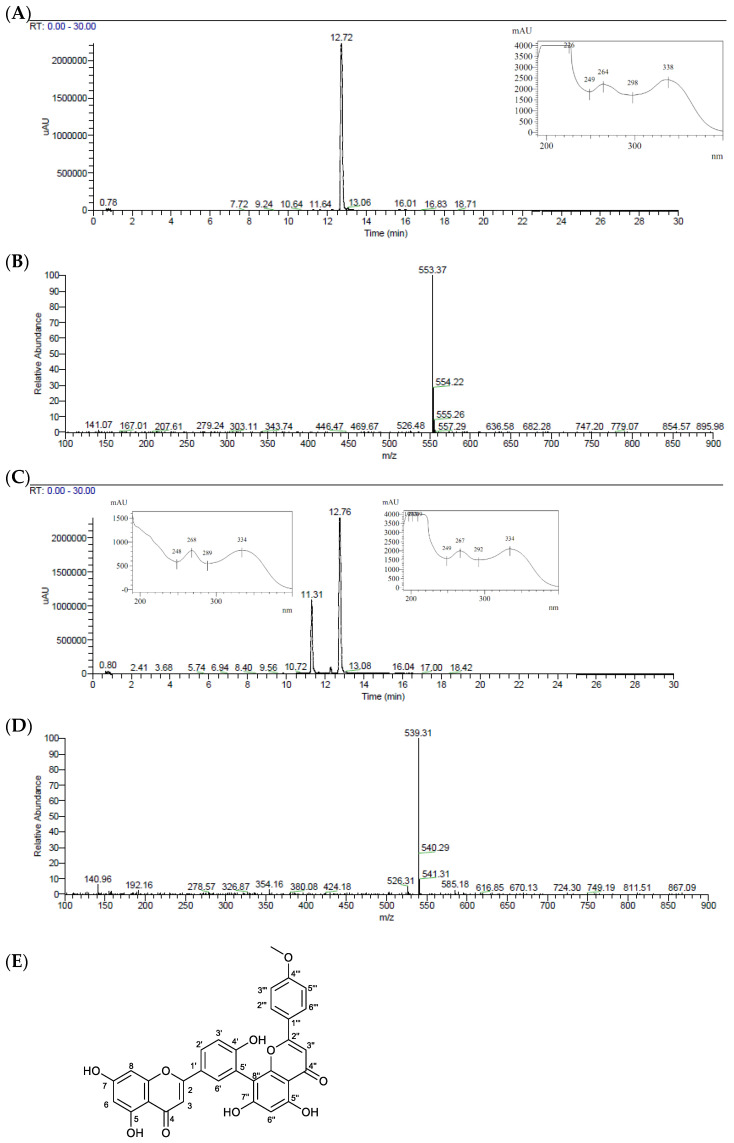
UPLC-MS/MS (254 nm) chromatogram, UV, and MS spectra of the F5 and F6 subfractions purified from dichloromethane fraction (DCM) of *Kielmeyera membranacea*. (**A**) F5 chromatogram and UV spectrum of the peak with t_R_ 12.72 min (podocarpusflavone A—PCFA). (**B**) F5 mass spectrum of the peak with t_R_ 12.72 min. (**C**) F6 chromatogram and UV spectra of the peak with t_R_ 11.31 and 12.76 min. (**D**) F6 mass spectrum of the peak with t_R_ 11.31 min. (**E**) PCFA structure.

*Kielmeyera membranacea* is a promising species with great antimycobacterial activity. In our earlier study, we found that the dichloromethane fraction (DCM) derived from the ethanol extract (EE) of *K. membranaceae* can inhibit both the avirulent *M. bovis* BCG and the virulent *M. tuberculosis* H37Rv strain. For this reason, the dichloromethane fraction was fractionated, and this process yielded six subfractions (F1–6), with the last two presenting the most promising antimycobacterial activity (Section 2.2). Therefore, F5 and F6 were analyzed by UPLC-MS/MS, and the compounds were identified based on their MS_n_ data and by comparison with MS databases and the literature (Figure 1A–D).

In the F5 chromatogram, only one peak was observed with t_R_ 12.72 min and a protonated molecule of *m*/*z* 553 [M+H]^+^, corresponding to C_31_H_20_O_10_ (Figure 1A,B). In the F6 chromatogram, two peaks were observed, the first with t_R_ 11.31 min and a protonated molecule of *m*/*z* 539 [M+H]^+^, corresponding to C_30_H_18_O_10_ (Figure 1C,D), probably corresponding to amentoflavone. The second peak with t_R_ 12.76 min was identified as the same flavonoid of F5 after comparing UV spectra and the MS fragmentation patterns. The compound isolated in F5 was further characterized by NMR spectroscopy as the biflavone podocarpusflavone A (PCFA) (Figure 1E), in accordance with the literature [19]. ^1^H and ^13^C NMR spectra are presented in the Supplementary Material Figure S1.

Podocarpusflavone A: ^1^H NMR (DMSO-*d*_6_, 500 MHz) δ 6.82 (s, H-3), 6.17 (*d*, *J* 1.8 Hz, H-6), 6.41 (*d*, *J* 1.8 Hz, H-8), 8.04 (*d*, *J* 2.2 Hz, H-2′), 7.11 (*d*, *J* 8.6 Hz, H-5′), 7.99 (*dd*, *J* 2.2; 8.6 Hz, H-6′), 6.88 (*s*, H-3″), 6.35 (*s*, H-6″), 7.70 (*br d*, *J* 8.9 Hz, H-2‴ and H-6‴), 6.89 (*br d*, *J* 8.9 Hz, H-3‴ and H-5‴), 3.73 (*s*, 4‴-OCH3), 12.89 (*s*, 5-OH), 13.09 (*s*, 5″-OH); ^13^*C NMR (DMSO-d_6_*, 125 MHz) δ 164.0 (C-2), 102.1 (C-3), 181.8 (C-4), 161.5 (C-5), 98.8 (C-6), 164.1 (C-7), 94.0 (C-8), 157.4 (C-9), 103.7 (C-10), 120.5 (C-1′), 131.4 (C-2′), 120.6 (C-3′), 160.6 (C-4′), 116.7 (C-5′), 127.7 (C-6′), 164.0 (C-2″), 103.3 (C-3″), 182.1 (C-4″), 160.6 (C-5″), 99.2 (C-6″), 163.1 (C-7″), 104.5 (C-8″), 154.6 (C-9″), 103.4 (C-10″), 123.1 (C-1‴), 128.1 (C-2‴), 114.5 (C-3‴), 162.2 (C-4‴), 114.5 (C-5‴), 128.1 (C-6‴), 55.5 (4‴-OCH_3_); ESI-MS *m*/*z* 553.37 [M+]+ (calculated for C_31_H_20_O_10_, 552.45). 

PCFA, also known as 4‴*O*-methylamentoflavone, is a dimer of apigenin and acacetin (4′-methyl ether derivative of apigenin) and has a C3′→C8″ interflavonoid linkage. Although PCFA has been described for some plant species belonging to the genera *Podocarpus* (Podocarpaceae) [20], *Garcinia* (Clusiaceae) [21], and *Juniperus* (Cupressaceae) [22], for the genus *Kielmeyera* there is only one report for *Kielmeyera variabilis* [19] and no reports of *K. membranacea*. Moreover, PCFA has exhibited anticancer [20,23], antileishmanial [24], antiviral [25,26], and antioxidant [19] activities. It can also inhibit NO production in LPS-stimulated macrophages [27] and NADPH oxidase and ROS production in stimulated human neutrophils [28].

### 2.2. Antimycobacterial Activity on Extracellular Bacteria and in Infected Human Macrophages

**Figure 2:**
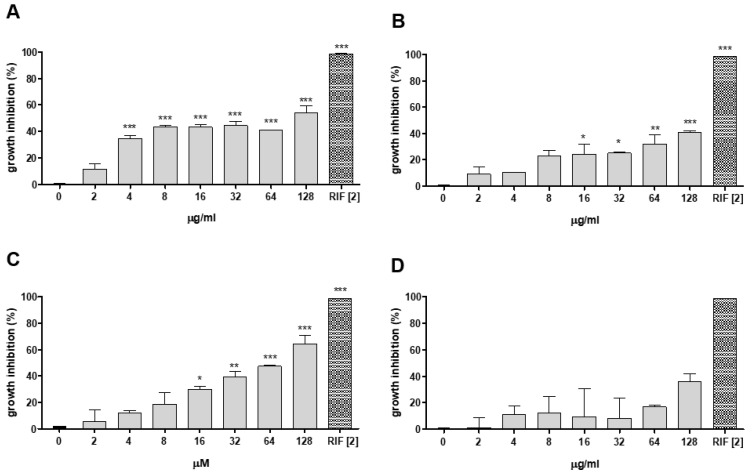
Growth inhibition of *Mycobacterium marinum* Wasabi by *Kielmeyera membranacea*. (**A**) ethanolic extract (EE); (**B**) dichloromethane fraction (DCM); (**C**) podocarpusflavone A (PCFA; F5); and (**D**) F6. Microplate assay with green-fluorescent bacteria after 5 days of incubation. Fluorescence reading was performed using excitation at 493 nm and emission at 509 nm. *M. marinum* treated with rifampicin (RIF) at a concentration of 2 µg/mL was used as a positive control, and untreated *M. marinum* was used as a negative control. *** *p* <0.001, ** *p* < 0.01, and * *p* < 0.05 when compared to the untreated group (0).

**Figure 3:**
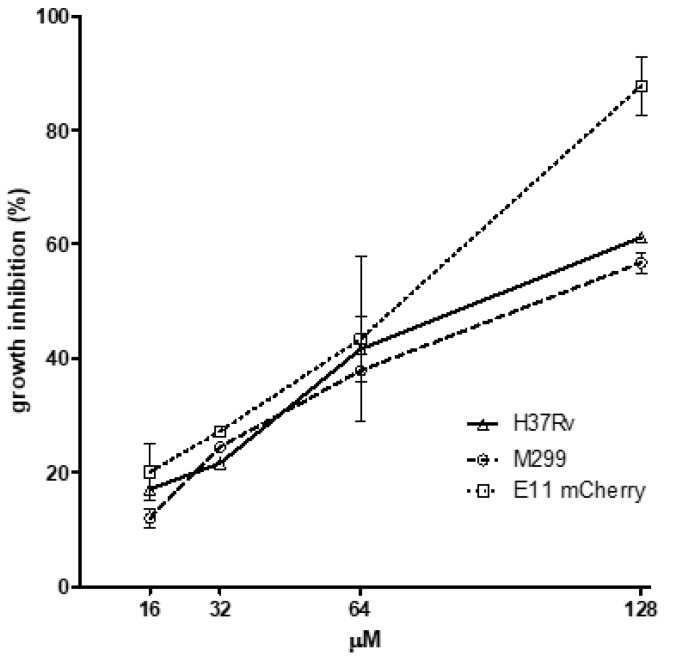
Growth inhibition of *Mycobacterium* strains by podocarpusflavone A (PCFA). Microplate assay after 5 days of incubation in the presence of PCFA (16–128 µM). Growth of *Mycobacterium tuberculosis* strains H37Rv and M299 was evaluated by the MTT test. The growth of *M. marinum* E11 mCherry red-fluorescent bacteria was assessed by measuring bacterial fluorescence (excitation at 588 nm and emission at 610 nm). The untreated *Mycobacterium* strains were used as a negative control, and rifampicin (RIF) was used as a positive control, with MIC values for each of the strains equal to 0.3 µg/mL for M299; 1 µg/mL for H37Rv and 4 µg/mL for E11 mCherry, respectively.

Aiming to select the best conditions for in vivo experiments, the minimum inhibitory concentration (MIC) of PCFA was evaluated in the *M*. *marinum* Wasabi strain. PCFA (F5) showed a MIC_50_ value of 34.38 ± 1.88 µM (equivalent to 18.99 ± 1.03 µg/mL) and was more active than the EE and DCM samples (MIC_50_ of 91.82 ± 1.62 and 125.1 ± 4.34 µg/mL, respectively) (Figure 2). The other fractions, F1-4 and F6, showed low inhibition of mycobacterial growth (Figure 2 and Supplementary Material Figure S2 for F1–4). PCFA has also been demonstrated to be effective against *M. marinum* E11 mCherry strain, *M. tuberculosis* strains H37Rv and M299 (hypervirulent), with MIC_50_ 34.83 ± 1.56, 87.38 ± 1.08 and 99.61 ± 1.03 µM, respectively, suggesting that PCFA is partially responsible for the activity of the DCM (Figure 3).

**Figure 4:**
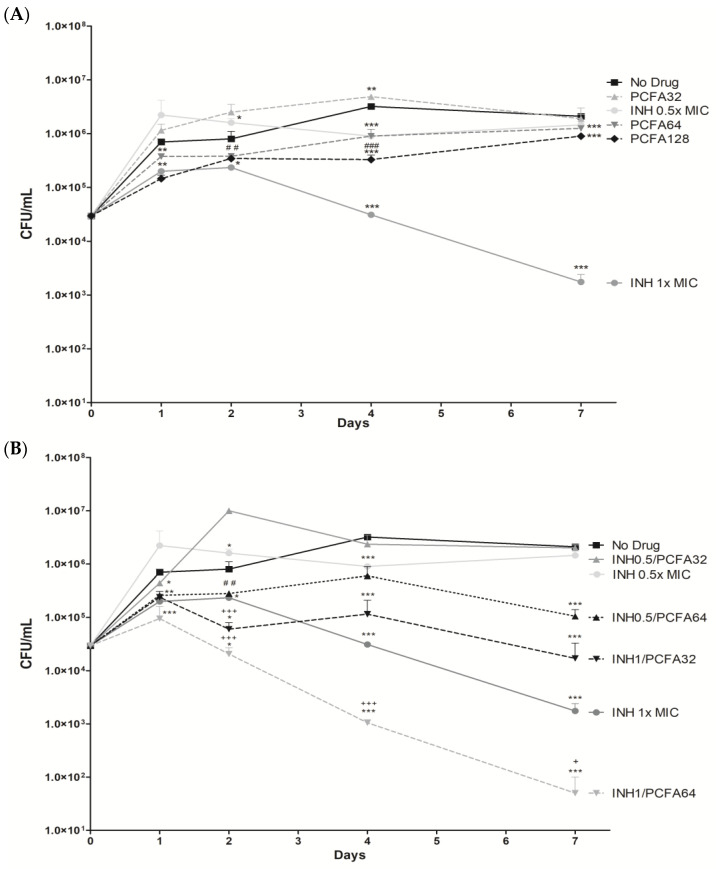
Effect of podocarpusflavone A (PCFA), isoniazid (INH), and INH-PCFA combinations on *Mycobacterium tuberculosis* H37Rv strain growth in THP-1-derived macrophages. (**A**) THP-1 macrophages were infected and treated with PCFA at 32, 64, and 128 µM and INH at 0.5× MIC (0.032 µg/mL) and 1× MIC (0.063 µg/mL). (**B**) THP-1 macrophages infected and treated with combinations of INH-PCFA. The intracellular viability of the bacteria was determined by CFU counting. Values were reported as mean ± error. The mean value of each group was significantly different from the mean value of the controls, as indicated in the graph after the two-way ANOVA analysis. *** *p* < 0.001, ** *p* < 0.01, and * *p* < 0.05 compared to no drug. ^###^ *p* < 0.001 and ^##^ *p* < 0.01 compared to INH 0.5× MIC. ^+++^ *p* < 0.001 and ^+^ *p* < 0.05 compared to INH 1× MIC.

The ability of PCFA, or isoniazid either on its own or in combination therapy with (INH), to inhibit the intracellular growth of mycobacteria was tested in THP-1-derived macrophages infected with *M. tuberculosis* H37Rv strain (Figure 4). This in vitro-infected macrophage model allows researchers to mimic the environment typically encountered by mycobacteria during infection [29]. The results demonstrated that PCFA alone at 64 and 128 µM was able to reduce bacterial growth when compared to untreated bacteria, as well as when compared to INH 0.5× MIC (0.032 µg/mL) at days 2 and 4 (Figure 4A). PCFA also reduced mycobacterial growth in combination with INH: INH 0.5× MIC + PCFA 64 µM was more efficient than INH 0.5× MIC alone. Likewise, INH 1× MIC + PCFA 32 µM and INH 1× MIC + PCFA 64 µM were more efficient than INH 1× MIC alone (0.063 µg/mL) (Figure 4B). Interestingly, the inhibitory effect was increased with the combined INH 1× MIC+ PCFA 64 µM, resulting in statistical significance on days 2, 4, and 7 (Figure 4B). Moreover, on days 4 and 7, the observed effect for the combination INH 1× MIC + PCFA 64 µM (Figure 4B) was higher than the sum of the single effects of INH 1× and PCFA 64 µM (Figure 4A). No significant toxicity was observed for PCFA on non-infected THP-1 macrophage culture, while it exhibited selectivity against the *M. tuberculosis* H37Rv strain. These findings indicate that further studies should be conducted to better characterize PCFA as a combination drug regarding novel combination regimens for TB treatment. In these multidrug regimens, a companion drug is used to prevent treatment failure due to acquired resistance to the core drugs. Some also help to reduce the risk of relapse [30].

INH is an essential first-line anti-TB drug with a well-described mechanism of action. In general, after activation by catalase-peroxidase (KatG), INH leads to the inhibition of enoyl acyl transporter reductase (InhA), which is involved in the biosynthesis of mycolic acids, essential constituents of the bacterial cell wall [31]. In this context, the mode of action of PCFA remains to be studied, and its prediction has been described in Section 2.5. However, the higher inhibitory effect of PCFA and INH together, when compared to the sum of their single effects, suggests that PCFA may act synergistically with INH.

### 2.3. In Vivo Toxicological Analysis

**Figure 5:**
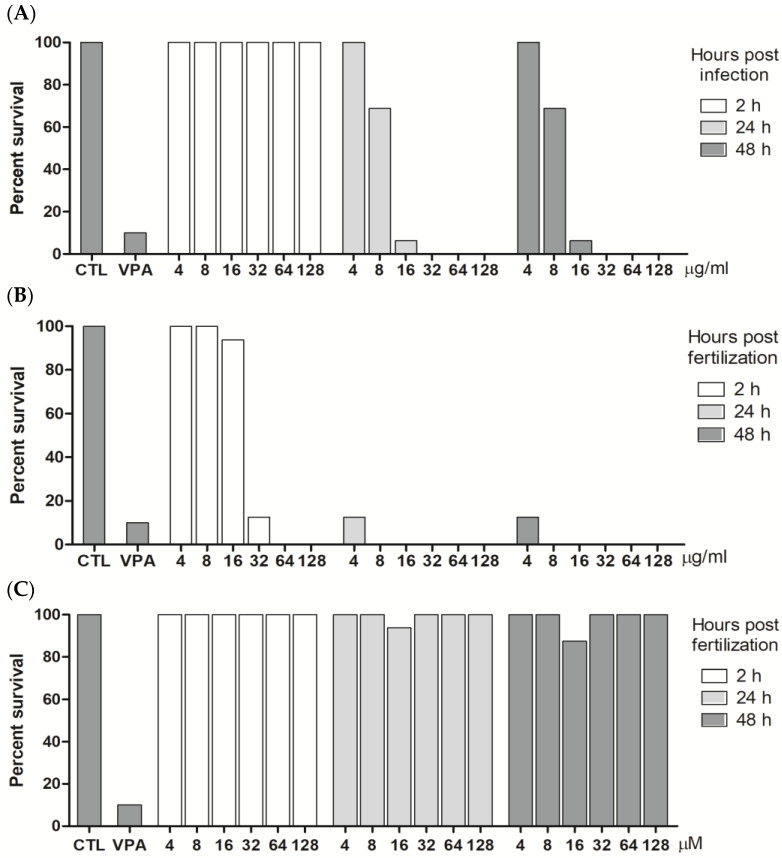
Viability of zebrafish larvae treated with *Kielmeyera membranacea* samples. The wild-type zebrafish larvae were treated with ethanolic extract (EE) (**A**), dichloromethane fraction (DCM) (**B**), and PCFA (**C**) for 2, 24, and 48 hpf, and viability was evaluated by microscopy-based morphological analysis. CTL (negative control, dilution water) and VPA (positive control, valproic acid—15 μM).

To determine the possible toxicity of the *Kielmeyera membranacea* samples in an in vivo model, zebrafish embryos were treated with EE, DCM (4–128 µg/mL), and PCFA (4–128 µM), and the embryos’ survival was examined by counting viable and dead larvae (Figure 5A–C). As can be seen in Figure 5, groups treated with EE (4, 8, 16, 32, 64, 128 μg/mL) showed no mortality (0%) at 2 h post-fertilization (hpf) (Figure 5A). These results should provide encouragement for researchers to conduct other pharmacological studies with *K. membranacea*, considering the good toxicological profile observed in vivo by the well-accepted fish embryo toxicity test (FET) with zebrafish (*Danio rerio*) [32]. PCFA showed no significant differences in embryo mortality compared to the control group (CTL) (Figure 5C). The group treated with valproic acid (VPA—15 μM), a zebrafish larvae control toxic drug, showed a high mortality rate (90%) (Figure 5A–C). The solvent used, DMSO (0.1% *v*/*v*), resulted in no fatalities (0% mortality rate at 2, 24, and 48 hpf).

Based on the antimycobacterial and toxicological results, a 32 µM dose was tested for in vivo efficacy against *M. marinum* infection. This dose did not result in any gross morphological change to embryonic development and was near the MIC_50_ of PCFA.

### 2.4. In Vivo Antimycobacterial Activity of Kielmeyera Membranacea Extract, Fraction, and Podocarpusflavone A Tested in the Zebrafish Mycobacterium marinum Infection Model

**Figure 6:**
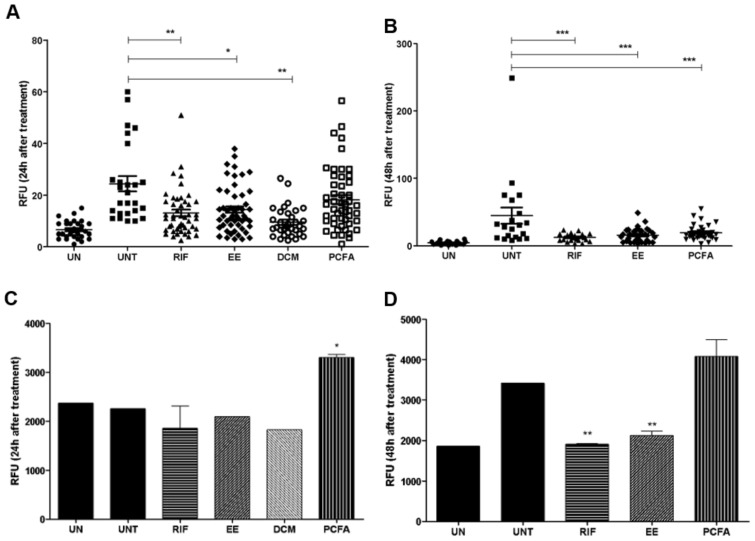
Effect of treatment with the ethanolic extract (EE), dichloromethane fraction (DCM) (32 µg/mL), and podocarpusflavone A (PCFA) (32 µM) on mycobacterial growth (mCherry E11) in 4- and 5-day-old Tg(mpeg1:eGFP)gl22 larvae after yolk sac injection of *Mycobacterium marinum*. (**A**) Red-fluorescent mycobacteria measured by COPAS analysis after 24 h, and (**B**) 48 h. (**C**) Green-fluorescent macrophages were measured by COPAS analysis after 24 h and (**D**) 48 h. UN: uninfected; UNT: untreated; RIF: rifampicin. *** *p* < 0.001 ** *p* < 0.01 and * *p* < 0.05 when compared to the infected and untreated group (UNT).

Initially, the antibacterial efficacy of the EE, DCM, and PCFA was assessed by monitoring the fluorescence intensity correlating with bacterial burden present either in the total body or only in the tail region of 5-day-old zebrafish larvae using COPAS analysis after yolk infection (Figure 6A,B). Larvae treated with the EE showed a reduction in the bacterial burden after 24 and 48 h. It is important to mention that, after 24 h, the EE and DCM showed similar in vivo activity despite the slightly higher in vitro extracellular antimycobacterial activity observed for the EE (Figure 2). The DCM was not evaluated at 48 h due to its toxicity. PCFA showed an inhibition trend after 24 h. However, after 48 h, it caused a statistically significant decrease in the bacterial burden, suggesting a time-dependent effect. Regarding macrophage fluorescence, it can be seen that PCFA significantly enhanced it after *M. marinum* infection at 24 and 48 h, which can be correlated with a high migration pattern associated with the infection site (Figure 6C,D).

**Figure 7:**
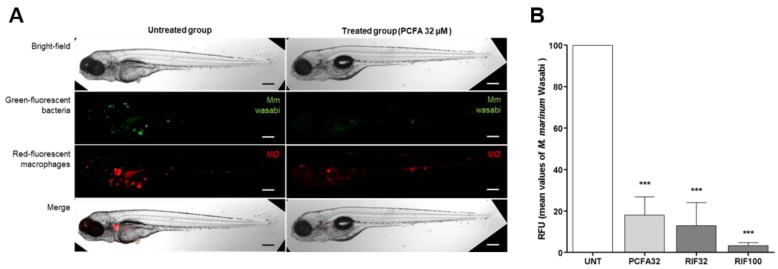
Caudal vein injection of *Mycobacterium marinum* and effect of treatment on infected larvae. Fluorescence microscopy images of fluorescent zebrafish and *M. marinum*. (**A**) Transgenic zebrafish larva with red fluorescent macrophages Tg(mpeg1:mCherry)gl23 infected with green fluorescent *M. marinum* (Wasabi), untreated or treated with PCFA (podocarpusflavone A) at 32 µM after 3 days. (**B**) Mean values of relative fluorescence units of green-fluorescent bacteria after 3 days with PCFA (32 µM) and rifampicin (RIF) (32 and 100 µM) treatment. Scale bar: 250 µm. *** *p* < 0.001 compared to the infected and untreated group (UNT).

At the final step of the in vivo study, larvae infected through the caudal vein by *M. marinum* microinjection were treated with PCFA at 32 µM for 5 days and refreshed every second day. Quantification of the systemic bacterial burden of *M. marinum*, through fluorescence imaging with repeated measurements of each larva, showed a statistically significant decrease at day 5 post-infection. Compared with the infected and untreated larvae at 5 dpi, there were fewer total infection foci and the areas of infection were smaller, clearly demonstrating the potential of PCFA to reduce (81.93 ± 8.76%) the bacterial burden in a biologically relevant setting (Figure 7). A similar effect was observed with rifampicin at the same concentration, 32 µM.

### 2.5. Podocarpusflavone: A Mode of Action Study

**Figure 8:**
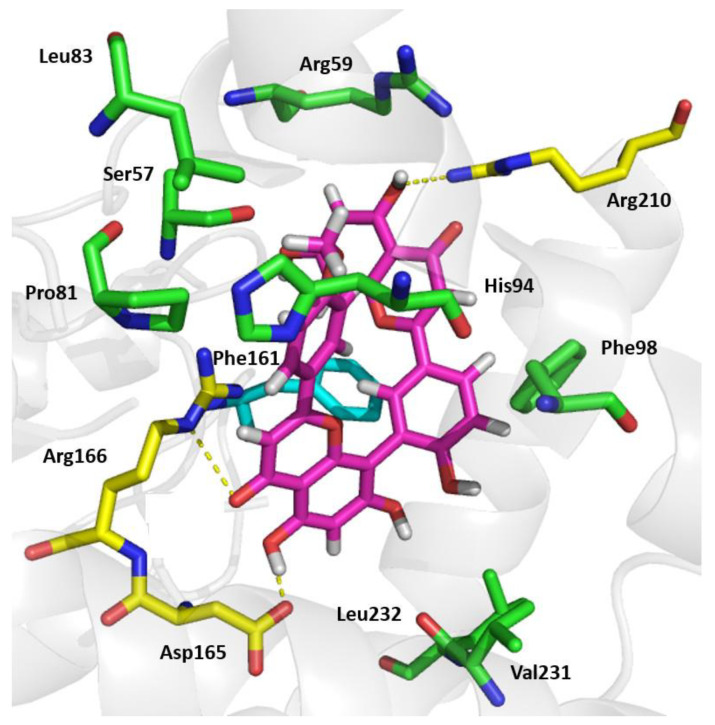
Molecular docking of podocarpusflavone A (PCFA) and PtpB of *Mycobacterium tuberculosis*. The carbon atoms of the molecule are colored pink at the ATP-binding site of PtpB. The amino acid residues in green show the interactions between van der Waals forces, the residues depicted in yellow are those involved in hydrogen bonding, π-π stacking is indicated in light blue, oxygen atoms are shown in red, and nitrogen atoms are shown in dark blue. Yellow dashed lines indicate hydrogen bonding. Picture made with Pymol, version 0.99.

**Figure 9:**
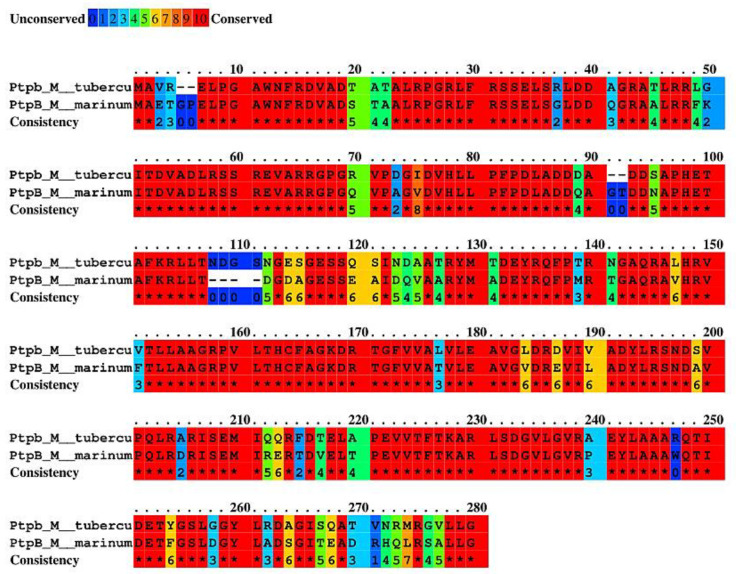
Amino acid sequence alignment of PtpB of *Mycobacterium tuberculosis* and *M. marinum*, * refers to the same amino acid residues in protein alignment, showing the consistency of the results. Image from the server Praline (https://www.ibi.vu.nl/programs/pralinewww/) accessed on 29 October 2024.

Chemogenomics studies were used to predict the potential mode of action of PCFA in *M. tuberculosis*. Protein tyrosine phosphatase B (PtpB), one of the virulence factors secreted into the host cell by *M. tuberculosis*, was predicted as the target responsible for the antimycobacterial activity of PCFA. Meaningful interactions for enzyme inhibition were observed via visual inspection (Figure 8). For instance, the molecule interacts with hydrogen bonds with residues Arg166 and Asp165 and achieves hydrophobic/aromatic interactions with the side chain of Phe161 with a geometry like the π stacking proposed for the polyphenol compound kuwanol E [33,34]. Besides kuwanol E, few PtpB inhibitors have been reported in the literature, as reviewed by Kuban-Jankowska [35]. Using Protein BLAST alignment protein sequence (http://blast.ncbi.nlm.nih.gov/Blast.cgi) accessed on 18 August 2023, we found that *M. tuberculosis* PtpB is 78% identical in the amino acid sequence and 84% identical in the catalytic domain in comparison to *M. marinum* PtpB (Figure 9).

Notably, PtpB prevents infected macrophage apoptosis by activating Akt and blocking caspase 3 activity [36]. Recently, it has been shown that PtpB inhibits gasdermin D (GSDMD)-dependent pyroptosis to promote *M. tuberculosis* intracellular survival in macrophages [37]. In addition, PtpB attenuates host immune defenses by interfering with signal transduction pathways in macrophages and, therefore, is a promising target for new anti-TB drug development [33,34,38]. Specifically, PtpB blocks the IFN-γ stimulated IL-6 production by downregulating ERK 1/2 and p38 activity [39]. IL-6 plays an important role in fighting *M. tuberculosis* infection, upregulating microbicidal activity in macrophages and initiating the systemic immune response [40]; its suppression contributes to *M. tuberculosis* intracellular survival. Consequently, the genetic inactivation of PtpB in *M*. *tuberculosis* accelerated mycobacterial cell death in activated macrophages and in the guinea pig model [39,41].

The prediction of PtpB as the target of PCFA concurs with the experimental results, considering that the observed activity of PCFA is promising, mainly with regard to macrophage and zebrafish infection. These results showed the importance of the reversion (or at least partial reversion) of the immune response inhibition modulated by PtpB. The combination of a PtpB inhibitor, which reduces *M. tuberculosis’* ability to evade immune responses, with a bactericidal antitubercular agent representing a highly promising strategy for TB treatment [35]. In this context, the activity observed using the combination of INH and PCFA (Figure 4) further corroborates this concept. Notably, no intrinsic roles have been identified between PtpB and the INH-related enzymes InhA and KatG.

### 2.6. Expression of Cytokines and Macrophage Markers in Zebrafish Infected with Mycobacterium marinum

Given that the in silico study predicted a potential interaction between PCFA and PtpB, which may be linked to the immune response, we evaluated the expression of cytokines. For this purpose, mRNA was obtained from the infected zebrafish larvae cells to characterize the profile of the host’s immune response in the acute phase of infection with mycobacteria. This was achieved through quantification of the relative expression of mRNAs responsible for encoding important antimycobacterial defense mediators by real-time qPCR. After 5 days of infection with *M. marinum*, the relative expression of mRNA of IL-10, IL-6, IL-12, TNF-α, and the chemokine receptor CXCR3 was measured in whole fish.

**Figure 10:**
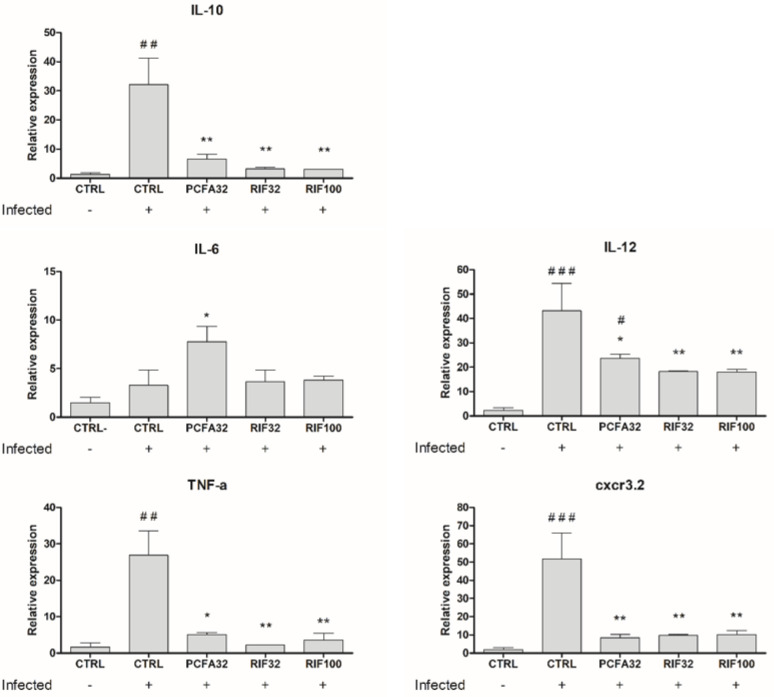
Expression levels of cytokines and macrophage markers in zebrafish larvae infected with *Mycobacterium marinum* determined by RT-qPCR. The Tg(mpeg1:mCherry)gl23 zebrafish larvae, infected (+) or non-infected (−) with *M. marinum*, were treated with podocarpusflavone A at 32 µM (PCFA32), rifampicin at 32 or 100 µM (RIF32 and RIF100) or were left untreated (CTRL) for 5 days, and relative expression of mRNA was measured for IL-12, IL-6, TNF-α, IL-10, and CXCR3.2. The expression of each target gene was related to the expression of the housekeeping ppial gene. The data were expressed as the mean ± SEM. * *p* < 0.05 and ** *p* < 0.01 compared to the CTRL infected group and # *p* < 0.05, ## *p* < 0.01 and ### *p* < 0.001 as compared to the CTRL non-infected group.

Consistent with the established role of inflammatory mediators in host defense against mycobacterial infection [42,43,44,45], the pro-inflammatory cytokines IL-12 and TNF-α expression were upregulated on day 5 post-infection in zebrafish larvae. After being treated with PCFA or rifampicin for 72 h, infected zebrafish larvae exhibited reduced transcriptional levels of the pro-inflammatory cytokines IL-12 and TNF-α, presumably indicating the resolution of the infection (Figure 10). In contrast, no statistically significant difference was observed for IL-6 between infected and non-infected controls. It is known that mycobacteria can modulate host IL-6 production, facilitating disease progression [40]. However, after PCFA treatment, a statistically significant increase was observed in the IL-6 mRNA level (Figure 10), which could be attributed to the inhibition of PtpB by PCFA, as predicted in silico, since PtpB blocks IL-6 production [39].

IL-10, the only anti-inflammatory cytokine evaluated in the present study, weakens the immune defense against *M. marinum* in infected zebrafish. Therefore, its levels are enhanced after infection as a mycobacterial survival immunomodulatory mechanism [46]. However, the treatment with PCFA or rifampicin reduced its levels due to infection control.

Macrophages are the primary cells of the host’s innate immune defense that interact with mycobacteria, and are stimulated to produce a variety of inflammatory mediators, like those described above: IL-6, IL-12, and TNF-α. Moreover, macrophages play a pivotal role in mycobacterial infections because they are motile and phagocytic cells while being a constituent cell type of the characteristic granulomas [47]. At the transcriptional level, infection in zebrafish triggers the expression of cxcr3.2 in macrophages, enhancing bacterial containment by increasing macrophage motility. This, in turn, facilitates macrophage-mediated bacterial dissemination and leads to a higher number of granulomas. Also, notably, cxcr3.2 is a functional homolog of human CXCR3 [48,49]. As shown in Figure 10, the cxcr3.2 level was enhanced due to the infection, and the treatment with PCFA and rifampicin attenuated its level; this can be directly associated with the infection resolution.

## 3. Materials and Methods

### 3.1. Reagents

Middlebrook 7H9 mycobacterial media was obtained from Difco (Detroit, MI, USA), while ADC supplements were sourced from BD Biosciences (Sparks, MD, USA). Rifampicin and MTT (3-(4,5-dimethylthiazol-2-yl)-2,5-diphenyltetrazolium bromide) were acquired from Sigma-Aldrich (St. Louis, MO, USA). Samples were dissolved in dimethyl sulfoxide (DMSO, Sigma Aldrich), whereas other reagents used for cell culture were dissolved in sterile phosphate-buffered saline (PBS) and sterilized by filtration.

### 3.2. General Experimental Procedures

^1^H and ^13^C NMR spectra of F5 (podocarpusflavone A) were obtained from 500 and 125 MHz, respectively, on a Varian MERCURY-VX 500 MHz. Size exclusion chromatography was performed on Sephadex LH-20. Eluates were monitored by thin-layer chromatography (TLC) on silica 60 F254 using chloroform–methanol (6:1), visualized under UV light, and revealed with phosphoric vanillin under heating. The fractions were analyzed by HPLC-DAD on a Shimadzu SCL-10A with Diode Array Detector SPD-M10A with absorptions measured from 200 to 600 nm.

### 3.3. Plant Material

Extract and fractions from leaves of previously obtained *Kielmeyera membranacea* Casar. (Calophyllaceae) [13] were used for the development of this research. Briefly, *K. membranacea* leaves were collected at Carapebus, Rio de Janeiro, Brazil. The plant material was identified by a botanist, Prof. Dr. Tatiana U. P. Konno, and a voucher specimen was deposited in the herbarium of UFRJ-RJ (RFA 38752). The leaves were then air-dried at room temperature, ground into a fine powder, and extracted through maceration with ethanol until fully exhausted. The crude extract (EE), obtained by rotary evaporation at 45 °C, was dissolved in H_2_O: MeOH (1:9), and fractions were obtained sequentially by liquid–liquid partition. Firstly, hexane was obtained, then, after MeOH removal, dichloromethane was obtained, resulting in the dichloromethane fraction (DCM). This research adhered to all applicable federal guidelines and institutional policies regarding the use of botanical material for research purposes (SISBIO/ICMBio No 62.455-11; SISGEN/MMA No: AAA989F).

### 3.4. Isolation and Characterization of Podocarpusflavone A

An aliquot of 1888 mg of the dichloromethane fraction was chromatographed on a Sephadex LH-20 gel column (GE Healthcare, Chicago, IL, USA) (60 × 5.5 cm; methanol), yielding 6 subfractions. After thin-layer chromatography (TLC) analysis, subfraction 5 (F5) was identified as an isolated compound 1 (101.9 mg). Compound 1 was identified as the biflavone podocarpusflavone A based on MS data (UPLC-MS/MS analysis), *m*/*z* 553.37 [M+H]+, and NMR spectroscopic data, in accordance with previous reports from the literature [20].

### 3.5. UPLC-MS/MS Analysis

The analysis of the subfractions obtained from the dichloromethane fraction was performed using a Dionex Focused Ultimate 3000 equipped with a DAD detector coupled with an LCQ Fleet Ion Trap Mass Spectrometer ThermoFisher Scientific (Waltham, MA, USA). The chromatographic conditions were an Ascentis^®^ Express C18 column (100 mm × 4.6 mm; 1.7 μm particle size) Supelco^®^ (Bellefonte, PA, USA) maintained at 25 °C, flow rate 0.2 mL/min, injection volume of 5 µL. The eluents were H_2_O (0.01% H_3_PO_4_) (A) and acetonitrile (ACN, B). The mobile phase elution was performed in the following gradient mode: 0–15 min, A–B (90:10→10:90); 15–20 min, A–B (10:90→0:100); 20–35 min, A–B (0:100→0:100); 35–37 min, A–B (0:100→90:10); 37–40 min, A–B (90:10→90:10), and 40 min as total time analysis. The mass spectrometry parameters were as follows: capillary voltage of 36 V, nebulization 5.5 Bar, drying gas flow of 10 L/min, drying temperature of 250 °C, and collision energy of 35 eV. Signal acquisition was performed in positive ion mode at *m*/*z* between 100 and 900 and MS2 of the base peak ions. The results were processed using XCalibur software ThermoFisher Scientific Version 2.0 (Waltham, MA, USA).

### 3.6. Bacterial Strain Preparation

The bacterial strains *Mycobacterium tuberculosis* H37Rv (a low virulent laboratory strain, ATCC 27294) and Beijing M299 (a highly virulent strain isolated from TB patient in Mozambique) were used for in vitro tests. Mycobacterial strains were cultured at 37 °C in Middlebrook 7H9 supplemented with 0.5% glycerol, 0.05% Tween-80, and 10% acid-albumin-dextrose-catalase (ADC). The bacterial strains *M. marinum* E11, expressing mCherry fluorescent protein and *M. marinum* Wasabi, expressing green fluorescent protein [49] were used for in vitro and in vivo tests inducing an infection by yolk or caudal vein in zebrafish embryos, respectively. Briefly, a colony of *M*. *marinum* was picked from 7H10 supplemented with 10% oleic acid-albumin-dextrose-catalase (OADC), and the colony was suspended in 7H9 supplemented with 10% ADC and cultured overnight at 28 °C. After 24 h, the OD at 600 nm (OD600) was measured (Eppendorf Biophotometer 6131, Eppendorf, Hamburg, Germany). Bacteria in the logarithmic growth phase were harvested and washed three times with sterile PBS. The inoculum was resuspended to a target concentration of 200 colony-forming units CFU/ηL in 2% polyvinylpyrrolidone-40 solution (PVP40) for both in vitro and in vivo infection tests [50].

### 3.7. In Vitro Antimycobacterial Activity

During the mid-logarithmic growth phase, bacterial suspensions were plated in a 96-well microplate at 1 × 10^6^ CFU/well. The samples were added at 128, 64, 32, and 16 μg/mL or μM (for PCFA), along with rifampicin at concentrations ranging from 0.0032 to 4 μg/mL. The plates were sealed and incubated at 32 °C or 37 °C for *M. marinum* and *M. tuberculosis*, respectively, and 5% CO_2_ for 5 days. After these periods, the *M. tuberculosis* cultures were incubated for 3 h with MTT solution (5 mg/mL) and then treated with lysis buffer (20% w/v SDS/50% dimethylformamide—DMF in distilled water, pH 4.7) for 18 h. The optical density was measured at 570 nm [51]. The *M. marinum* fluorescent cultures were analyzed by fluorescence using 588 nm excitation and 610 nm emission for the E11 strain, and 493 nm excitation and 509 nm emission for the Wasabi strain. Mycobacteria treated and untreated with rifampicin were used as positive and negative controls, respectively.

### 3.8. Cytotoxicity and Infection of Macrophages

THP-1 macrophage cells were cultured and differentiated into macrophages with 50 ng/mL phorbol 12-myristate 13-acetate (PMA; Sigma-Aldrich). After 24 h, PMA was removed, and cells were treated with the samples. After 24, 48, 72, and 144 h, cell viability was measured using MTT. Then, to evaluate the sample’s intracellular antimycobacterial activity, following differentiation, the cells were infected with *M. tuberculosis* H37Rv strain at a multiplicity of infection (MOI) of 1:10. After 3 h of infection, extracellular bacteria were further removed by washing with warm PBS. Fresh medium with different treatments was added (isolated compound at 32, 64, and 128 μM, isoniazid (INH) at 0.5× and 1× MIC, 0.063 µg/mL, and their combinations). Macrophages were lysed at 0 h and 1, 2, 4, and 7 days after infection, plated on 7H10 agar plates, and incubated at 37 °C to determine total CFU after 10–14 days [52,53].

### 3.9. Zebrafish Husbandry

Zebrafish were maintained and handled in accordance with international consensus protocols [54]. The design and execution of all experiments complied with European regulations, in which zebrafish embryos and larvae were not considered experimental animals throughout our experiments (0–5 days post-fertilization [dpf]). Adult wild-type, Tg(mpeg1:eGFP)gl22 and Tg(mpeg1:mCherry)gl23 zebrafish were kept in glass aquaria (max 6 L^−1^, volume 10 L, 120 × 220 × 490 mm; Fleuren & Nooijen BV, Nederweert, The Netherlands) with circulating water (27.7 °C ± 0.1 on a 14 h/10 h light/dark cycle, lights on at 08:00). Artemia or Gemma Micro/Diamond (Skretting, Nutreco NV, Amersfoort, The Netherlands) were used for adult zebrafish feeding, and the water quality was monitored with a JUMO ACQUIS. For breeding, the zebrafish were kept overnight, and in the morning, the dividers between males and females were removed to collect fertilized eggs. Eggs, embryos, and larvae were kept in E3 medium (60 μg/mL^−1^ Instant Ocean Sea Salts [Sera, Heinsberg, Germany] in demineralized water, daily refreshed) at 28 °C.

### 3.10. Toxic Effect on Zebrafish Larvae

The wild-type zebrafish were maintained and handled, and the eggs were prepared as described in the previous section. The tests were performed in accordance with the fish embryo toxicity guide [55] and the scheme proposed by Lammer [32]. Ten wells of a 24-well plate were assigned to each sample concentration of treatment and negative and positive controls. A fertilized egg was added to each well with 2 mL of fish water solution. The plates were incubated at 28 °C, and the toxic effect was evaluated by analyzing the morphological patterns of zebrafish development recorded at 2, 24, and 48 h post-fertilization (hpf).

### 3.11. Inhibition of Mycobacterium marinum Growth by Yolk Infection

The Tg(mpeg1:eGFP)gl22 zebrafish embryos at 2 to 4 hpf were anesthetized with 0.02% (*w*/*v*) of ethyl 3-aminobenzoate (tricaine) buffer 10 min before injection and injected with 1 nL of 50 CFU/nL *M. marinum* E11 mCherry strain using the microinjection system (FemtoJet, Eppendorf, Hamburg, Germany). The Petri plates (92 × 16 mm) with 50 embryos were incubated at 28 °C. After 3 days, infected larvae were analyzed using COPAS™ (Complex Object Parametric Analyzer and Sorter) to check the established infection. Waterborne treatment with compounds commenced, and larvae were kept in E3 medium with compounds during the entire treatment at 28 °C. COPAS analysis and imaging were performed at 24 and 48 h post-treatment.

### 3.12. Inhibition of Mycobacterium marinum Growth by Caudal Vein Infection

The Tg(mpeg1:mCherry)gl23 zebrafish embryos were dechorionated manually at 24 hpf. Embryos of 30 hpf were anesthetized with 0.02% (*w*/*v*) of tricaine buffer 10 min prior and injected with 1 nL of 200 CFU/nL *M. marinum* Wasabi strain into the caudal vein at the blood island [41]. After injection, the larvae were kept at 28 °C overnight to check the establishment of the infection. Then, the embryos were transferred to 24-well plates (Greiner Bio-One) with compound solutions at 32 µM in an E3 medium. The waterborne treatment with compounds was refreshed every 48 h. For the duration of treatment, larvae were kept at 28 °C in an E3 medium with compounds until imaging or sampling took place. Quantification of the bacterial burden within individual larvae was analyzed through fluorescence imaging at 3 days post-treatment (dpt) and 4 days post-infection (dpi) to assess individual early bactericidal responses. The group size was designed at *n* = 20, and rifampicin at 32 and 100 µM was used as a positive control.

### 3.13. RNA Isolation, cDNA Synthesis, and RT-qPCR

Pools of twenty Tg(mpeg1:mCherry)gl23 larvae were homogenized in 1 mL of TRIzol reagent (Life Technologies, Carlsbad, CA, USA). Subsequently, total RNA was extracted and purified using RNeasy MinElute Cleanup Kit (Qiagen, Hilden, Germany), according to the manufacturer’s instructions. cDNAs were synthesized from 200 ng total RNAs, and qPCR was performed by using the iScript™ cDNA Synthesis Kit (Bio-Rad, Hercules, CA, USA) and iQ™ SYBR Green Supermix (Bio-Rad), according to the manufacturer’s protocols. RT-qPCR was conducted using the MyiQ Single-Color Real-Time PCR Detection System (Bio-Rad). The cycling started with a 3 min 95 °C pre-denaturation, followed by 40 cycles of 15 s 95 °C denaturation, 30 s annealing at 60 °C, and elongation for 30 s at 72 °C, with 0.5 °C increments every 10 s. For every sample, the threshold cycle (Ct) value was determined from the Ct value of a non-infected control sample, and the fold change of gene expression was calculated and normalized against the expression of the housekeeping gene ppial. The results were analyzed using the ∆∆Ct method [56].

### 3.14. Statistical Analysis

GraphPad Prism 5.0 and 10 were used for statistical analysis. One-way analysis of variance (ANOVA) with Tukey’s post-test or two-way analysis of variance (ANOVA) were used to evaluate paired replicates. The *p*-values of less than 0.05 were considered significant. IC_50_, MIC_50,_ and CC_50_ values were calculated by non-linear regression analysis of log[concentration]/inhibition curves by GraphPad Prism 5 Software applying a sigmoidal dose–response variable slope curve fitting using the different percentage obtained for each corresponding concentration in triplicate and were expressed as means with corresponding 95% confidence intervals.

### 3.15. In Silico Prediction of Podocarpusflavone A Mode of Action (MOA)

The active compound PCFA was drawn in ChemAxon’s MarvinSketch v. 6.2.1, saved in sdf format and then standardized with ChemAxon’s Standardizer v. 6.2.1. After this step, the compound was saved in Excel in the smiles molecular format. The smiles notation was then saved in csv format, and the biological targets were predicted using the command “python predict_binary.py input.csv 30 0.5” using Pidgin chemogenomics software v. 2 [57,58].

### 3.16. Molecular Docking Studies

GOLD v. 5.535 was applied to conduct the molecular docking studies using chain A of the 3D structure of the *M. tuberculosis* target protein tyrosine phosphatase B (PtpB), PDB code 2OZ5 [33], obtained by X-ray diffraction with 2 Å of resolution in a complex with its inhibitor (oxalylamino-methylene)-thiophene sulfonamide (OMTS). The protonation states of charged residues from podocarpusflavone A were defined at pH 7.4 using PROPKA 3.0 available on the PDB2PQR server [59]. Before docking studies, redocking of the inhibitor OMTS was performed with GOLD v.5.535, using ChemPLP, GoldScore, and ChemScore scoring functions, with search radii of 10, 15, and 20 Å, respectively, using the carbon atom #1130 belonging to Phe161 as a reference. After redocking, the best function and radius were chosen for the lowest value of Root Mean Square Deviation (RMSD) between the inhibitor (OMTS) removed from the crystal and the top pose obtained by the redocking methodology. Hermes software, available in Gold Suite (GOLD v. 5.535), was used to calculate the RMSD values. The 3D structure of podocarpusflavone A was constructed in Spartan’10 (Wavefunction Inc., Irvine, CA, USA), and the prediction of the molecular ionization states in the physiological pH was carried out with ChemAxon’s MarvinView, version 6.2.1 (http://www.chemaxon.com) accessed on 9 August 2023. Finally, the compound was energy-optimized in Spartan’10 using the B3LYP/6-31G * density functional method [60]. Analysis of the interactions between podocarpusflavone A and PtpB was performed in Pymol v. 0.99 (The PyMOL Molecular Graphics System, Schrodinger, LLC, New York, NY, USA) and Discovery Studio v. 16.1.0 (BIOVIA, Discovery Studio Visualizer, 2015).

## 4. Conclusions

Our study demonstrated the promising in vivo antimycobacterial activity of the *Kielmeyera membranacea* extract, displaying a favorable in vivo toxicological profile. We isolated and characterized podocarpusflavone A (PCFA) as an active compound in this extract. Intracellularly, PCFA reduced *Mycobacterium tuberculosis* H37Rv strain growth, particularly when combined with isoniazid. In addition, PCFA was active in two in vivo zebrafish larvae *M. marinum* infection models, which was a stimulating finding. Specifically, when the systemic infection was established by bacterial microinjection via the caudal vein, the efficacy of PCFA was comparable to that of rifampicin at 32 µM. In silico predictions further suggest that PCFA interacts with mycobacterial protein tyrosine phosphatase (PtpB), potentially blocking *M. tuberculosis*’s key immune evasion mechanism. This finding corroborates the positive modulation of IL-6 in vivo and the enhanced intracellular in vitro and in vivo antimycobacterial activity observed for PCFA. In short, our findings support further mammalian studies to explore the anti-TB potential of PCFA.

## Data Availability

Data are contained within the article and Supplementary Materials.

## Supplementary Materials

The following supporting information can be downloaded at https://www.mdpi.com/article/10.3390/ph17121560/s1, Figure S1. NMR spectra of the biflavone podocarpusflavone A (PCFA); Figure S2. Growth inhibition of Mycobacterium marinum Wasabi by subfractions of dichloromethane fraction from Kielmeyera membranacea.
