# Supplementary material for: Exploring the Antimycobacterial Potential of Podocarpusflavone A from Kielmeyera membranacea: In Vitro and In Vivo Insights
Source: Pharmaceuticals (Basel). 2024 Nov 21;17(12):1560. doi: 10.3390/ph17121560 (PMC11676425; doi:10.3390/ph17121560)
Supplement: Supplementary file 1 [file pharmaceuticals-17-01560-s001.zip › pharmaceuticals-3255061-supplementary.pdf]

SUPPLEMENTARY MATERIAL

Exploring the Antimycobacterial Potential of Podocarpusflavone A from *Kielmeyera membranacea*: In Vitro and In Vivo Insights

Marlon Heggdorne de Araujo [1,2], Salomé Muñoz Sánchez [2], Thatiana Lopes Biá Ventura Simão [3], Natalia Nowik [2], Stella Schuenck Antunes [1,4], Shaft Corrêa Pinto [1], Davide Sorze [5], Francesca Boldrin [5], Riccardo Manganelli [5], Nelilma Correia Romeiro [4], Elena B. Lasunskaia [3], Fons J. Verbeek [6], Herman P. Spaink [2] and Michelle Frazão Muzitano [1,*]

[1] Laboratório de Produtos Bioativos (LPBio), Instituto de Ciências Farmacêuticas, Universidade Federal do Rio de Janeiro, Campus Macaé, 27930-560, Macaé, RJ, Brazil; marlon.heggdorne@gmail.com (M.H.A.); stellantunes@yahoo.com.br (S.S.A.); shaftcp@yahoo.com.br (S.C.P.)

[2] Department of Animal Sciences and Health, Institute of Biology (IBL), Leiden University, 2333BE, Leiden, The Netherlands; s.munoz.sanchez@biology.leidenuniv.nl (S.M.S.); h.p.spaink@biology.leidenuniv.nl (H.P.S.)

[3] Laboratório de Biologia do Reconhecer (LBR), Centro de Biociências e Biotecnologia, Universidade Estadual do Norte Fluminense Darcy Ribeiro, 28013-602, Campos dos Goytacazes, RJ, Brazil; thativentura@yahoo.com.br (T.L.B.V.S.); elena@uenf.br (E.B.L.)

[4] Laboratório Integrado de Computação Científica (LICC), Universidade Federal do Rio de Janeiro, Campus Macaé, 27930-560, Macaé, RJ, Brazil; nelilmaromeiro@gmail.com (N.C.R.)

[5] Department of Molecular Medicine, University of Padova, 35121, Padova, Italy; davide.sorze@studenti.unipd.it (D.S.); francesca.boldrin@unipd.it (F.B.); riccardo.manganelli@unipd.it (R.M.)

[6] Leiden Institute of Advanced Computer Science, Leiden University, 2333CA, Leiden, The Netherlands; f.j.verbeek@liacs.leidenuniv.nl (F.J.V.)

* Correspondence: mfmuzitano@macae.ufrj.br or mfmuzitano@gmail.com; Tel.: 55-22-2141-4029

SUPPLEMENTARY FIGURES

A

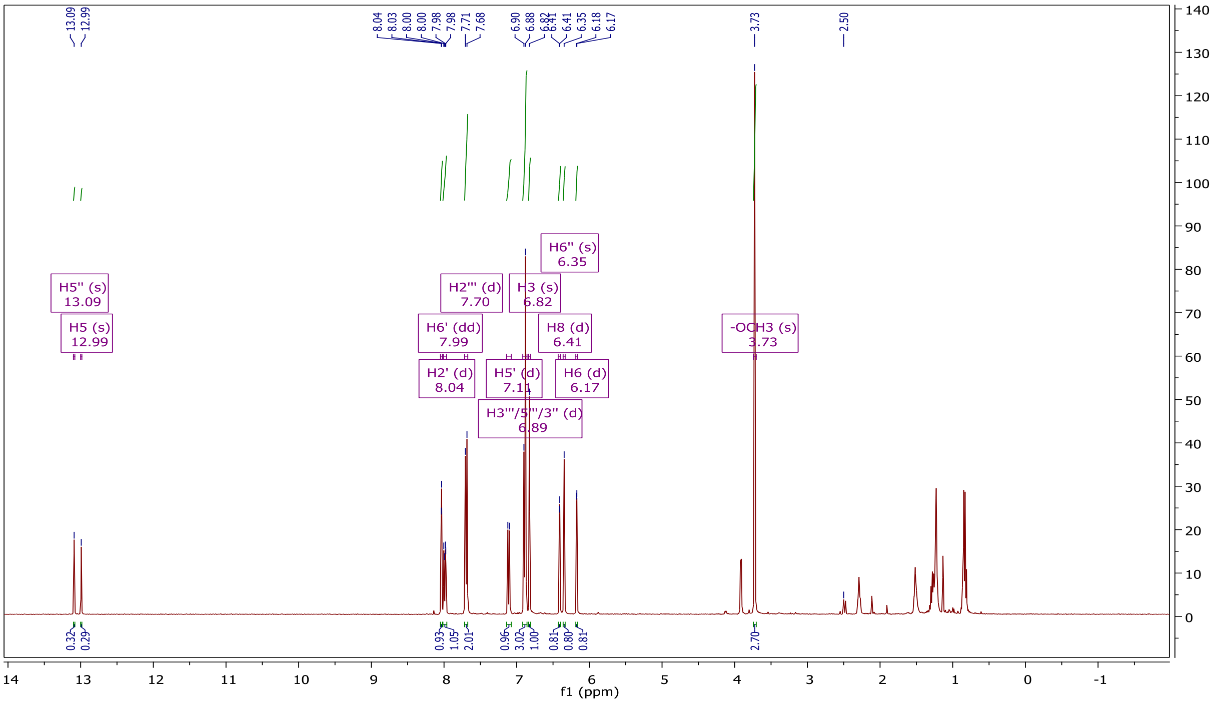

B

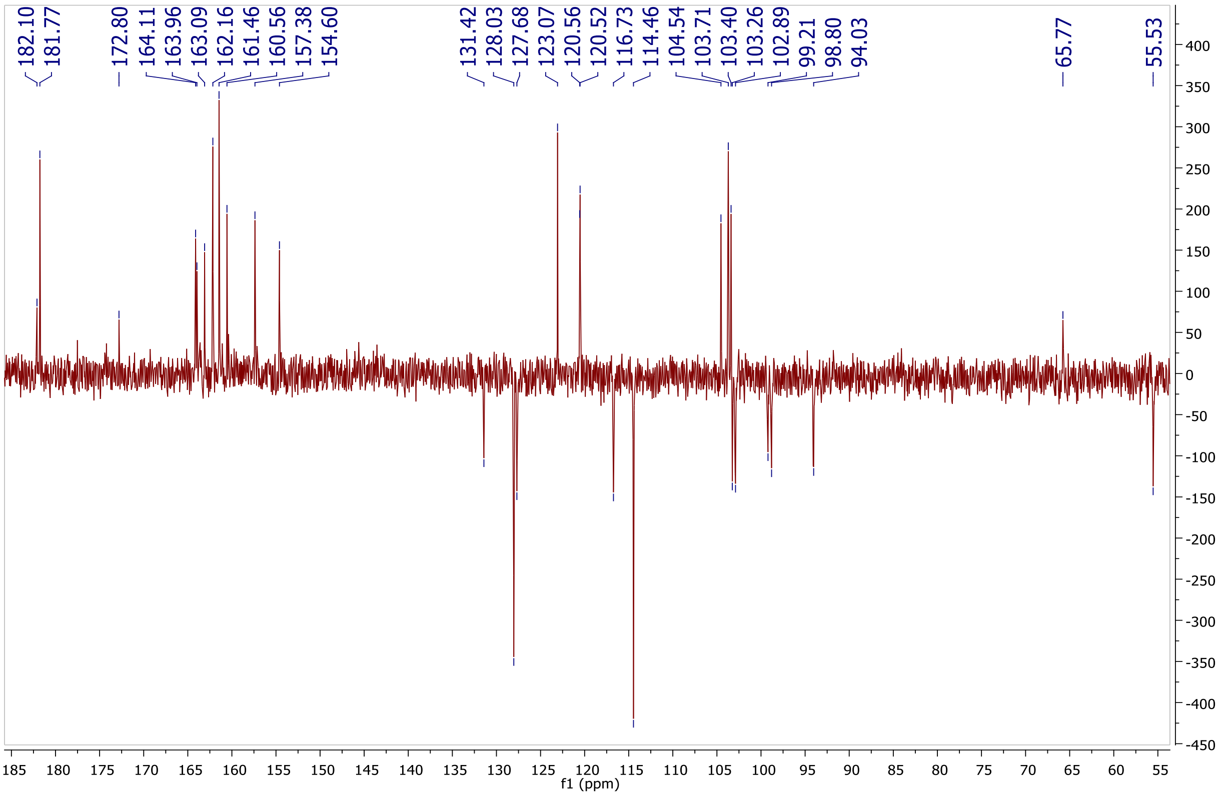

Figure S1. NMR spectra of the biflavone podocarpusflavone A (PCFA), (**A**) ^{1}H NMR spectrum (DMSO-d_6, 500 MHz); and (**B**) ^{13}C APT (DMSO-d_6, 125 MHz).

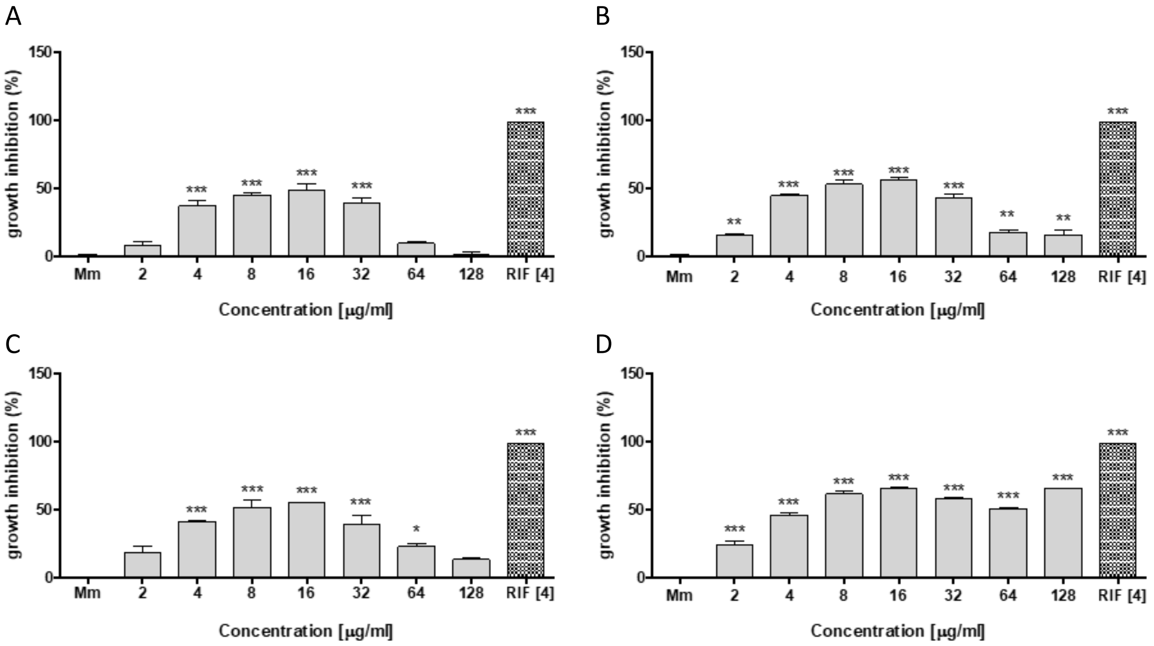

Figure S2. Growth inhibition of *Mycobacterium marinum* Wasabi by subfractions of dichloromethane fraction from *Kielmeyera membranacea*. (**A**) F1; (**B**) F2; (**C**) F3; and (**D**) F4. Microplate assay with green-fluorescent bacteria after 5 days of incubation. Fluorescence reading using excitation at 493 nm and emission at 509 nm. *M. marinum* treated with rifampicin (RIF) at a concentration of 4 µg/mL was used as positive control and untreated *M. marinum* was used as negative control. *** p <0.001, ** p < 0.01, and * p < 0.05 when compared to untreated group (Mm).
